# Temperature and livestock grazing trigger transcriptome responses in bumblebees along an elevational gradient

**DOI:** 10.1016/j.isci.2022.105175

**Published:** 2022-09-22

**Authors:** Kristof Brenzinger, Fabienne Maihoff, Marcell K. Peters, Leonie Schimmer, Thorsten Bischler, Alice Classen

**Affiliations:** 1 Department of Animal Ecology and Tropical Biology, Biocenter, University of Würzburg, 97074 Würzburg, Germany; 2 Core Unit Systems Medicine, University of Würzburg, 97080 Würzburg, Germany

**Keywords:** Biological sciences, Evolutionary biology, Evolutionary ecology, Omics

## Abstract

Climate and land-use changes cause increasing stress to pollinators but the molecular pathways underlying stress responses are poorly understood. Here, we analyzed the transcriptomic response of *Bombus lucorum* workers to temperature and livestock grazing. Bumblebees sampled along an elevational gradient, and from differently managed grassland sites (livestock grazing vs unmanaged) in the German Alps did not differ in the expression of genes known for thermal stress responses. Instead, metabolic energy production pathways were upregulated in bumblebees sampled in mid- or high elevations or during cool temperatures. Extensive grazing pressure led to an upregulation of genetic pathways involved in immunoregulation and DNA-repair. We conclude that widespread bumblebees are tolerant toward temperature fluctuations in temperate mountain environments. Moderate temperature increases may even release bumblebees from metabolic stress. However, transcriptome responses to even moderate management regimes highlight the completely underestimated complexity of human influence on natural pollinators.

## Highlights

• Upregulation of energy metabolism pathways in *Bombus lucorum* with increasing elevation
• Genes known for thermal stress responses did not change with increased elevation
• *Bombus lucorum* are tolerant toward relatively broad temperature fluctuations
• Grazing lead to an upregulation in genetic information processes in *B. lucorum*

## Graphical abstract

**Figure:**
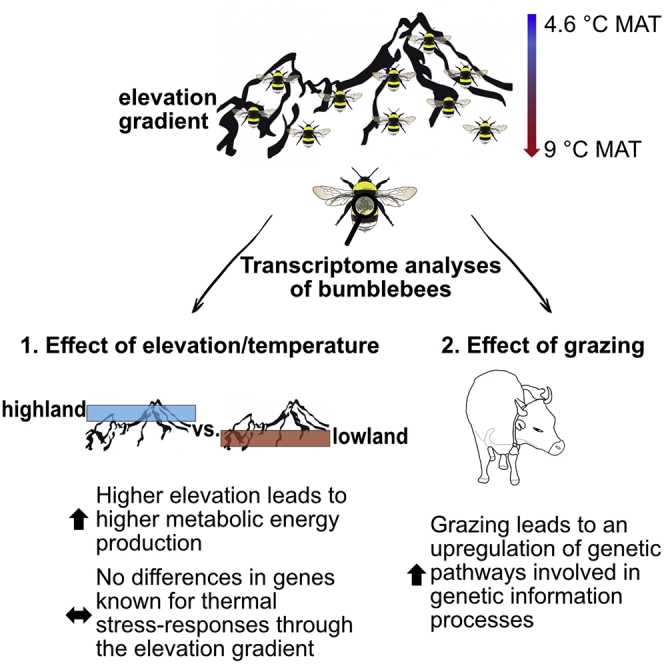

## Introduction

Countering the ongoing biodiversity crisis in times of global change is one of the major challenges of our time. Insects, accounting for a large proportion of terrestrial biodiversity, play a crucial role in this crisis. As ectothermic and small organisms, they are particularly sensitive to climatic changes (Halsch et al., 2021; Paaijmans et al., 2013) and tremendously suffer from the large-scale depletion of microhabitats and local resources through agricultural intensification and landscape homogenization (Habel et al., 2019; Wagner et al., 2021). An increasing number of studies have reported dramatic declines in insect populations, biomass, and richness worldwide (Hallmann et al., 2017; Seibold et al., 2019; Wagner et al., 2021). Importantly, such declines in species abundances or even local extinctions of populations are always the last step in the response of an insect population to global change drivers. Signals of environmental stress, however, may already be detectable on a molecular level (Hoffmann and Willi, 2008; Somero, 2005). For example, changes in gene expressions in response to changing environmental factors can help to identify the relevant stressors and to improve our understanding of the mechanisms that underlie population declines, So far, such transcriptome responses to global change drivers remain poorly understood (Dunning et al., 2014; Keller et al., 2013).

Species with large geographical ranges, for example, bumblebees, can encounter considerable environmental heterogeneity and are excellent models to shed light on genetic variation in relation to abiotic (e.g. temperature) and biotic (e.g. resources) environments (Pimsler et al., 2020). Bumblebees (Hymenoptera: Apidae: *Bombus*) are cold-adapted, large-bodied wild bees with primitively eusocial lifestyles, whose extant lineages started to diversify around 34 Mya during a period of substantial global cooling (Hines, 2008). On a genus level, they encompass wide geographic ranges, with diverse hotspots in both cool mountainous regions and high temperate latitudes (Williams, 1998), but also with several species in the Neotropics and Southeast Asia (Michener, 2007). In temperate mountains like the Alps, many, but not all, species have broad elevational distributions and cope with climatically heterogeneous environments (Sponsler et al., 2022).

One likely reason, why widespread bumblebees can cope with a variety of temperature regimes is that bumblebees are facultative endotherms, i.e. they can actively increase their body temperature by shivering their flight muscles (Dzialowski et al., 2014; Heinrich, 1972). Together with their relatively large body size and dense hair (Maebe et al., 2021a), this ability allows bumblebee individuals to forage at different temperatures below 5°C (Corbet et al., 1993) or even closer to the freezing point (Heinrich and Vogt, 1993). Physiologically, heat is generated by the burning of ATP, requiring the antagonistic interplay of the two enzymes fructose diphosphatase and phosphofructokinase (Heinrich, 1974, 1979; Surholt et al., 1990). Thus, along temperature gradients, such as mountain slopes, genes related to muscle function, thermogenesis, and substrate cycling can be expected to show expression patterns that reflect the increasing need for heat generation with decreasing temperatures.

Alternatively, genes associated with the critical thermal limits might show distinct expression patterns along temperature gradients. Workers of the same species (Pimsler et al., 2020) or queens of different subspecies (Maebe et al., 2021a) have already been shown to differ in their critical thermal minima (CTmin), depending on the temperature regime of their place of origin. Local adaptations in CTmin were linked to differential transcriptome responses after experimental cooling. This indicates that the limits of phenotypic plasticity can become genetically fixed or at least be transferred to the next generation (e.g. via epigenetic processes, such as DNA-methylation), which could facilitate species’ ability to occupy broad geographic ranges, by reducing the costs of plasticity in CTmin. Importantly, critical thermal maxima (CTmax) are species-specific (Martinet et al., 2021) and do neither show associations with local climates within species (Pimsler et al., 2020) nor across subspecies (Maebe et al., 2021a), suggesting that responses to warming temperatures are more constraint.

In line with relatively conserved critical thermal maxima, many bumblebee species are shifting their range toward cooler habitats regarding changing climate, either by retracting the trailing (warm) edge of their distribution (Fourcade et al., 2019), or by shifting upwards along elevational gradients (Biella et al., 2017; Marshall et al., 2019; Pyke et al., 2016). The variable extents to which bumblebee species perform uphill shifts might be subject to their species-specific capacity to cope with changing environmental conditions along the elevational slope (Marshall et al., 2020; Pyke et al., 2016). Whereas some temperature extremes may be offset at the individual or colony level through behavioral temperature regulation (Vogt, 1986), most bumblebees are expected to exhibit specific plastic responses, selection in various key traits, and/or range contractions under even the mildest climate change (reviewed in Maebe et al., 2021b). Several studies suggested that the expression of genes coding heat shock proteins (hsp) and their activators (aha) allow bumblebees to adapt to heat stress (Blasco-Lavilla et al., 2021; Liu et al., 2020b; Pimsler et al., 2020). Other genes involved in immune response, like cytochrome P450, rac1, and flotillin (Liu et al., 2020b; Pimsler et al., 2020), seem to play an additional role in response to stressing temperatures. However, these effects were so far only observed under extreme climatic conditions in climate chamber experiments or extreme environments (Tibetan Plateau). Whether these molecular responses explain compensations of sensitivity to hyperthermal stress (Martinet et al., 2021; Zambra et al., 2020), thus enabling the dispersal of species widely distributed in Europe in the first place, or whether they are involved in the responses along the elevational gradient is unknown.

Besides climate change, land use change is believed to be the strongest stressor for pollinators. In the Alpes, livestock grazing by cattle is an important component of the cultivated landscape and even part of the protection program in strongly protected areas, as it can increase e.g. plant diversity, but is highly dependent on the amounts of grazing mammals (Milchunas et al., 1988; Olff and Ritchie, 1998). The potential impact of cattle grazing on bumblebee fitness is little understood, but could affect wild bees directly or indirectly through various mechanisms, including effects on bee nesting and floral resource composition (Augustine and McNaughton, 1998; Carvell, 2002; Moreira et al., 2019; Roulston and Goodell, 2011; Sjödin, 2007). Additionally, grazing may fundamentally change soil, nectar and pollen characteristics by introducing new nitrogen substrates (Potts et al., 2015) (Ceulemans et al., 2017; Pers-Kamczyc et al., 2020) Even though little is known about the impact of grazing on pollinators, the substantial change in bumblebee’s environment alone likely causes variation in gene expression.

**Figure 1:**
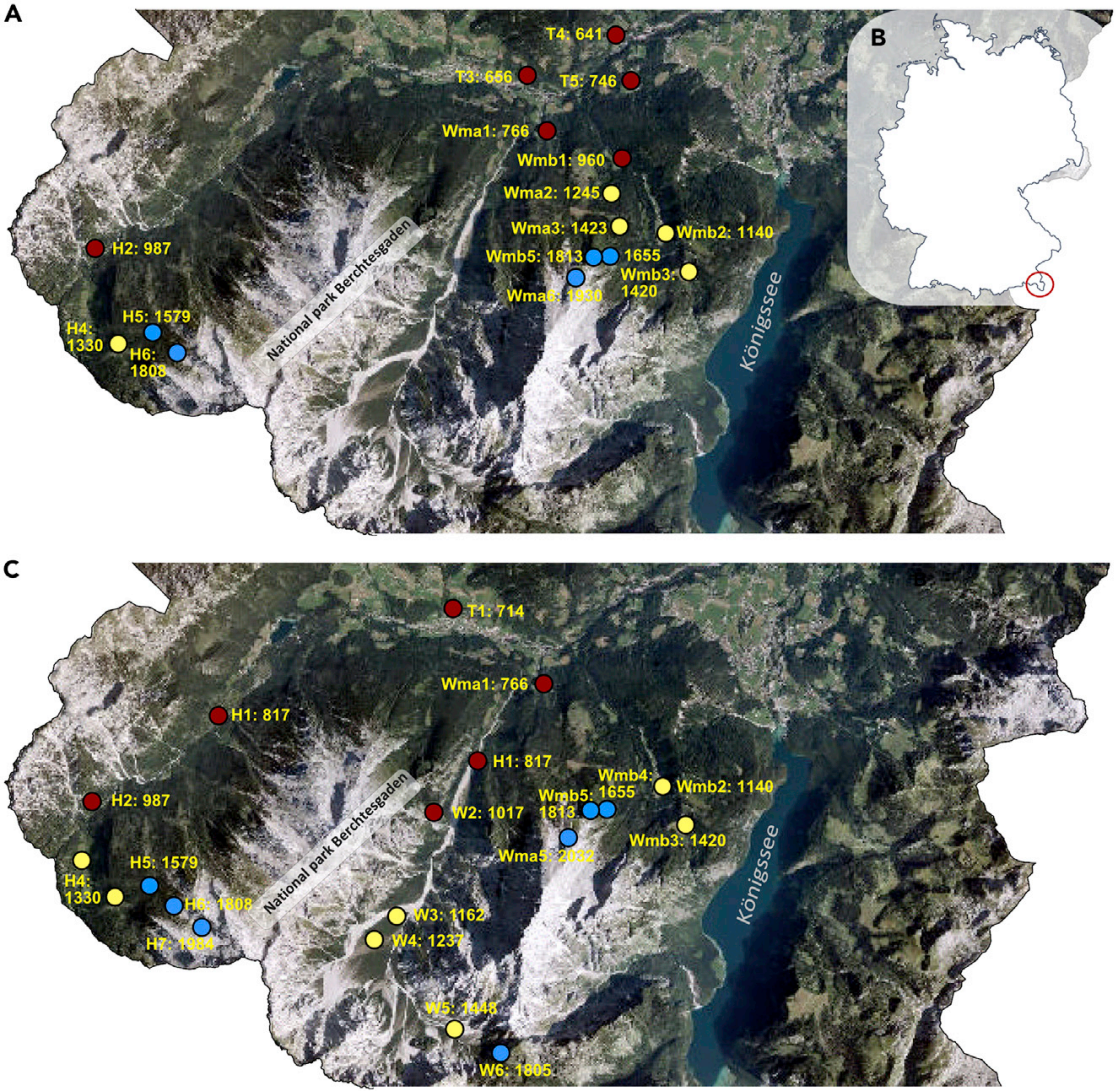
*B. lucorum* sample sites Bumblebee sample sites for the transcriptomic analyses (A) and the measurement of thermal tolerance (C), collected in and outside the Nationalpark Berchtesgaden, which is located in the south-eastern tip of Bavaria, Germany (B). Study sites covered an elevational range from 641 to 2,032 m a.s.l. (elevation given for each study site in meters above sea level). Dot color signals the elevational band (red = lowlands, yellow = mid-elevations, blue = highlands), which was used as the factor in the transcriptome analyses.

In this study, we aim to identify common regulatory factors that contribute to the (plastic) adaptation of bumblebees to different climates and farming systems. For this, we focus on the study of transcriptional changes in *B. lucorum*. On the one hand, this species occurs along an elevational gradient of 1400m, corresponding to a temperature gradient from 9 to 4.6°C mean annual temperature (a 0.5°C decrease per 100 m difference in elevation), and is also widely distributed over a large geographical area in Europe (Rasmont et al., 2015). Studies along an elevational gradient enable the prediction of long-term ecological responses to climate change using space-time substitution scenarios (Körner, 2007; Mayor et al., 2017). On the other hand, the species is considered more cold-adapted compared with its sister species *Bombus terrestris* (Geue and Thomassen, 2020) and is likely to be displaced by the latter under climate change (Herbertsson et al., 2021). It was further observed to spread to higher elevations (Pyke et al., 2016). With this, *B. lucorum* is a suitable model organism to observe plastic transcriptome changes related to temperature changes and may lead to predictions about how widespread bumblebees may adapt to changing climate. In addition, *B. lucorum* is a ground-nesting bumblebee species that is likely to suffer the most from grazing livestock. First, we examine the transcriptomic response along the elevation gradient (Figure 1A). To verify that elevational patterns in the transcriptome can be assigned to the change in temperature and not to co-varying factors (e.g. dropping partial pressure of respiratory gases, radiation, or vegetation), we, second, compared gene expression patterns from the elevational gradient with gene expression differences between individuals that were sampled during warm and cool days, but within the same elevational belt. In parallel, we examine critical thermal limits along the elevational gradient (Figure 1B). Finally, we analyzed the effect of livestock grazing on gene expression, as an alternative or additional stressor along the elevational slope. Overall, this approach allows us to identify regulatory factors that contribute to adaptation to global change stressors for bumblebees in temperate climates.

## Results

### *Bombus lucorum* gene expression patterns along elevation

**Figure 2:**
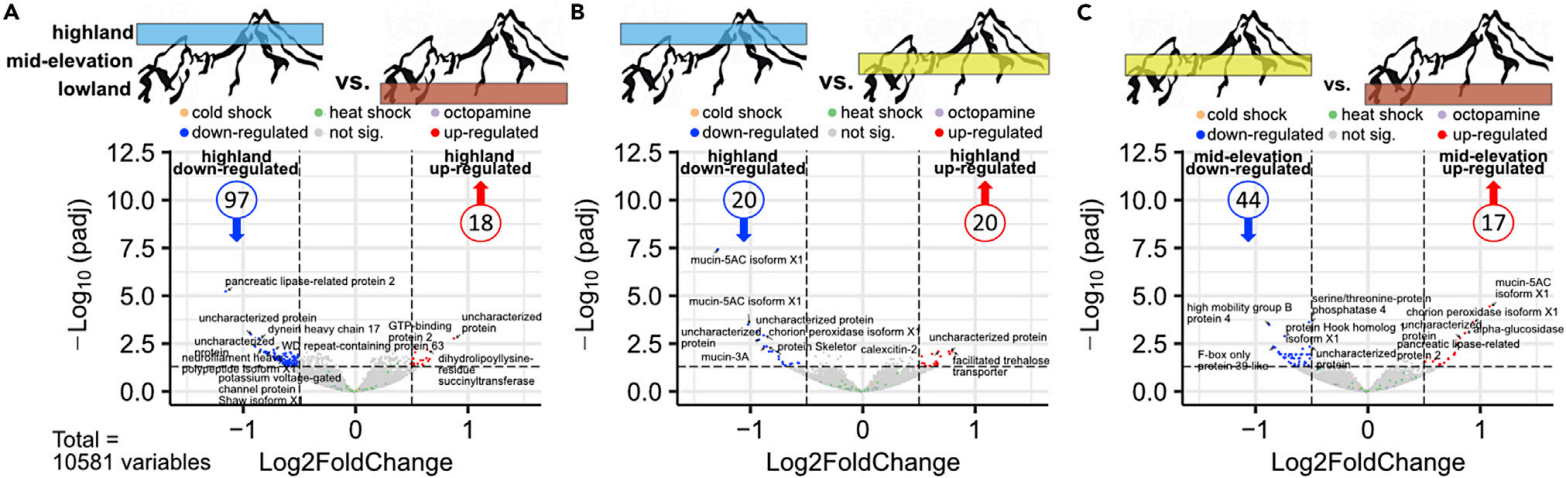
RNA-seq expression patters between different elevation belts Summary of the RNA-seq expression patterns by volcano plots obtained from DESeq2 analysis (*x*-axis represents the log2 of the fold change between the gene expression of two groups; the *y*-axis represents the negative decade logarithm of the adjusted *p*-value) for the three different comparisons between *B. lucorum* samples from the different elevation groups: highland vs lowland (A); highland vs mid-elevation (B); mid-elevation vs lowland (C). Red and blue points mark the genes with significantly increased or decreased expression respectively for the different group comparisons regarding always the first mentioned elevation group as up-regulated (reddish colors) and down-regulated (bluish colors). Most significant expressed genes were labeled with their name. Selected genes known to be involved in elevational adaption from other studies are highlighted in ochre (genes coding for cold shock protein), green (genes coding for heat shock protein) and purple (genes coding for octopamine protein), which all lay in the group of not significant regulated genes. Dashed lines represent applied significance thresholds.

**Figure 3:**
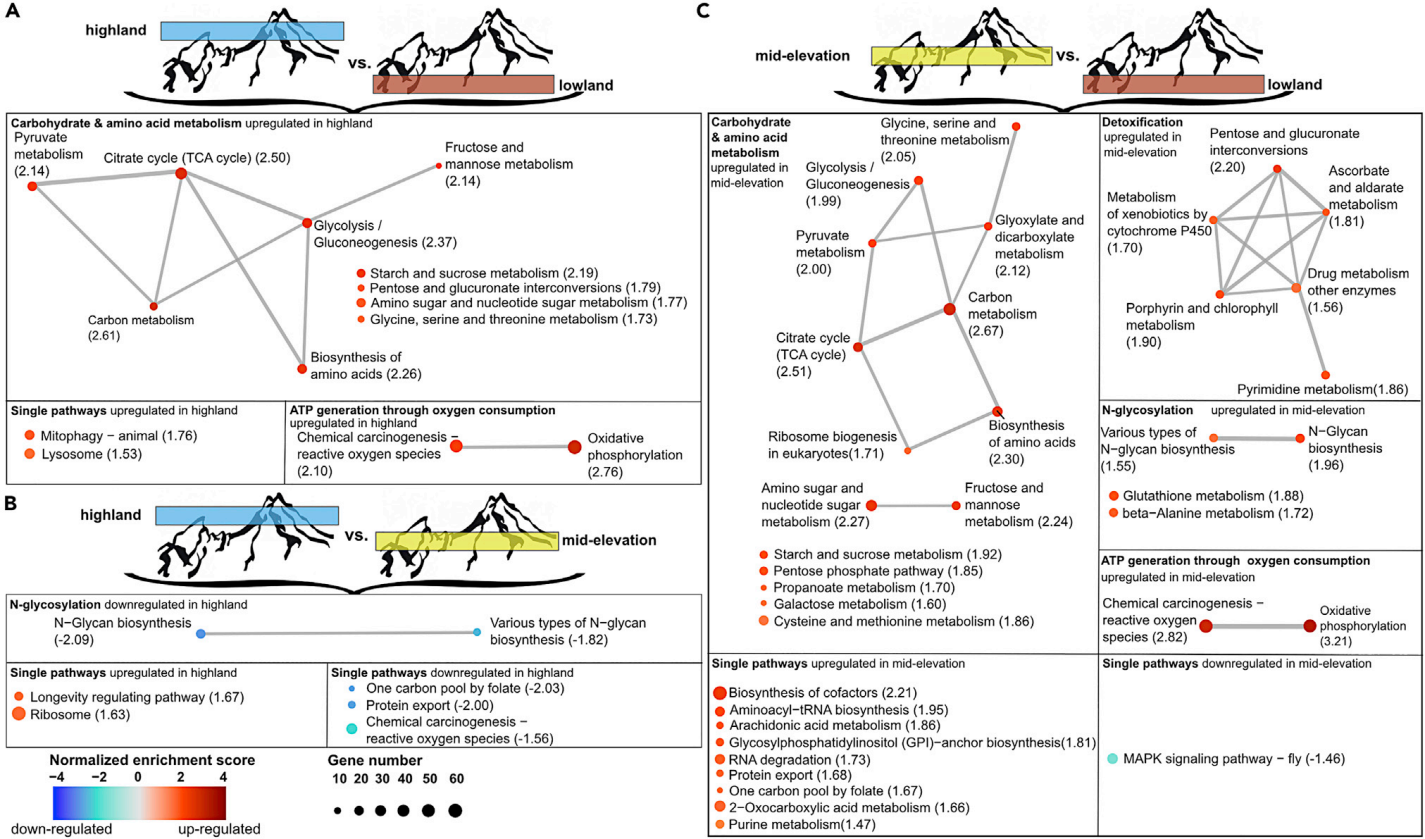
Gene set enrichment analysis (GSEA) between the different elevation belts Enrichment map representation of the ranked GSEA results obtained for two-way comparative analysis between *B. lucorum* samples from each elevation gradient group: highland vs lowland (A); highland vs mid-elevation (B); mid-elevation vs lowland (C). Enrichment maps from GSEA showing gene sets in an interaction network with nodes of transcripts received by RNA-seq with moderately conservative statistical significance (*p* < 0.005, FDR < 0.05, and overlap coefficient = 0.2). Clustered sub networks of nodes (depicted as dotted circles) reflect generic functional networks. Nodes represent enriched gene sets, where node size corresponds to the number of genes and color intensity corresponds to normalized enrichment score. Edges represent overlap between gene sets with line thickness correlating to the degree of overlap. Pathways were manually grouped into different categories based on their KEGG assignment.

We analyzed the expression of 10,581 protein-coding genes based on RNA-seq data (Table S4). When equally splitting the elevational gradient and comparing these elevation belts with each other, we detected 18 and 20 upregulated genes in the highlands compared with lowlands and mid-elevations, respectively (Figures 2A and 2B). The mid-elevation area showed 17 and 20 upregulated genes compared with lowland and highland, respectively (Figures 2B, 2C, and Table S5). The greatest amounts of upregulated genes were observed for the bumblebees from lowland sites with 44 vs mid-elevation and 97 vs highland (Figures 2A, 2C, and Table S5). A check for genes that are important in temperature or elevation stress responses (calumenin, flotillin, cytochrome P450, chaperone DnaJ, BAG proteins, phosphofructokinase, heat shock proteins, cold shock proteins) (Liu et al., 2020a, 2020b; Pimsler et al., 2020; Sivan et al., 2017) did not show a significant change between the elevational belts (Figures 2, S1 and S2). One-third of the significantly regulated genes do not currently have annotation in the reference bumblebee (*B. terrestris*) genome (Table S5), and the other significantly regulated genes did not show a clear association with a particular functional group of enzymatic pathways. However, if we analyzed the different pathways based on the log2fold change via GSEA analyses, which considers all genes involved in one pathway in parallel, we observed an upregulation from mid-elevation and highland *B. lucorum* samples of the carbon metabolism (Glycolysis/Gluconeogenesis, Fructose and mannose metabolism, Pyruvate metabolism, TCA cycle, Glycine, serine and threonine metabolism, starch and sucrose metabolism) and oxygen transformation pathways compared with lowland bumblebees (Figures 3A, 3B, and Table S6). Additionally, in mid-elevation bumblebees N-glycosylation pathways were upregulated compared with lowland and highland bumblebees, whereas detoxification pathways were upregulated compared with lowland bumblebees (Figures 3A, 3C; Table S6). Overall, the transcriptome from bumblebees sampled in mid-elevation was more like the transcriptome from highland samples, than from lowland samples. Even though we find more single downregulated genes in mid-elevation and highland when compared with lowlands (Figure 2 and Table S5), these genes could not be grouped into specific pathways (Figure 3 and Table S6).

Importantly, elevation and mean annual temperature (MAT) are strongly correlated (Persons *R* = 0.99). Accordingly, grouping the elevational gradient in lowlands, mid-elevations and highlands equals a categorization in three different MAT categories (warm, medium, cold), and produces the same results.

### *Bombus lucorum* gene expression patterns under low and high sampling temperature

**Figure 4:**
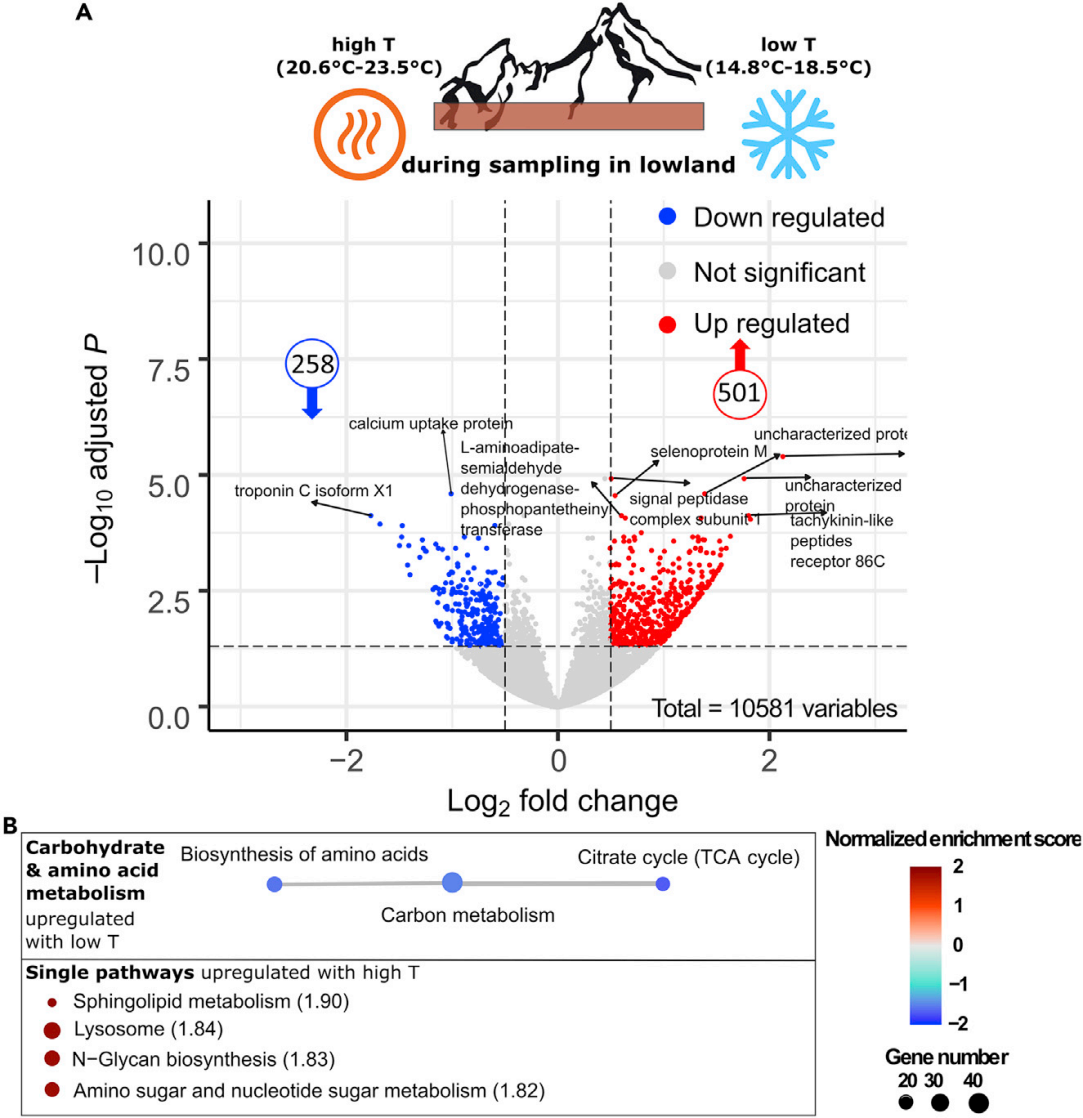
Summary of the RNA-seq results between *B. lucorum* sampled at high and low temperatures in lowland Summary of the RNA-seq results by volcano plot (A) obtained from DESeq2 analysis (*x*-axis represents the log2 of the fold change, *y*-axis represents the negative decade logarithm of the significance value) and Enrichment Map representation of the ranked GSEA results (B) between high and low temperatures during sampling of *B. lucorum* in lowland with moderately statistical significance (*p* < 0.1, FDR < 0.1, and overlap coefficient = 0.2). A detailed description of the different shown parameters can be obtained from Figures 2 and 3.

To verify that the upregulation of the carbon metabolism and oxygen transformation pathways in bees sampled in mid- and highlands compared with lowland samples is a response to the different temperature regimes, we investigated transcriptomic responses to different sampling temperatures. We assume that those will show similar patterns if temperature is the driving force. Indeed, when splitting the lowland samples into lower (14.8–18.5°C) and higher (20.6–23.5°C) environmental temperature during sampling, we observed a similar trend for bumblebee samples during low sampling temperature as for the higher elevation samples. Parts of the carbon cycle (TCA cycle) were upregulated under lower sampling temperatures (Figure 4B and Table S8). In these analyses, we found 501 single expressed genes up-, and 258 down-regulated when bumblebees were collected during higher sampling temperatures and compared with bumblebees collected during lower sampling temperatures (Figure 4A and Table S7). Around 25% of these up- and down-regulated genes originate from uncharacterized proteins. Within the downregulated genes at high temperatures, 15 expressed genes could be linked to the fatty acid metabolism and 11 transcripts to the carbon metabolism based on KEGG annotation. From the upregulated genes during high sampling temperatures, 11 expressed genes can be grouped together in the formation of lysosomes, 11 genes belonged to amino sugar and nucleotide sugar formation, and 8 genes were involved in neuroactive ligand-receptor interaction.

### Bumblebee CT_min_ and CT_max_ along the elevation gradient

**Figure 5:**
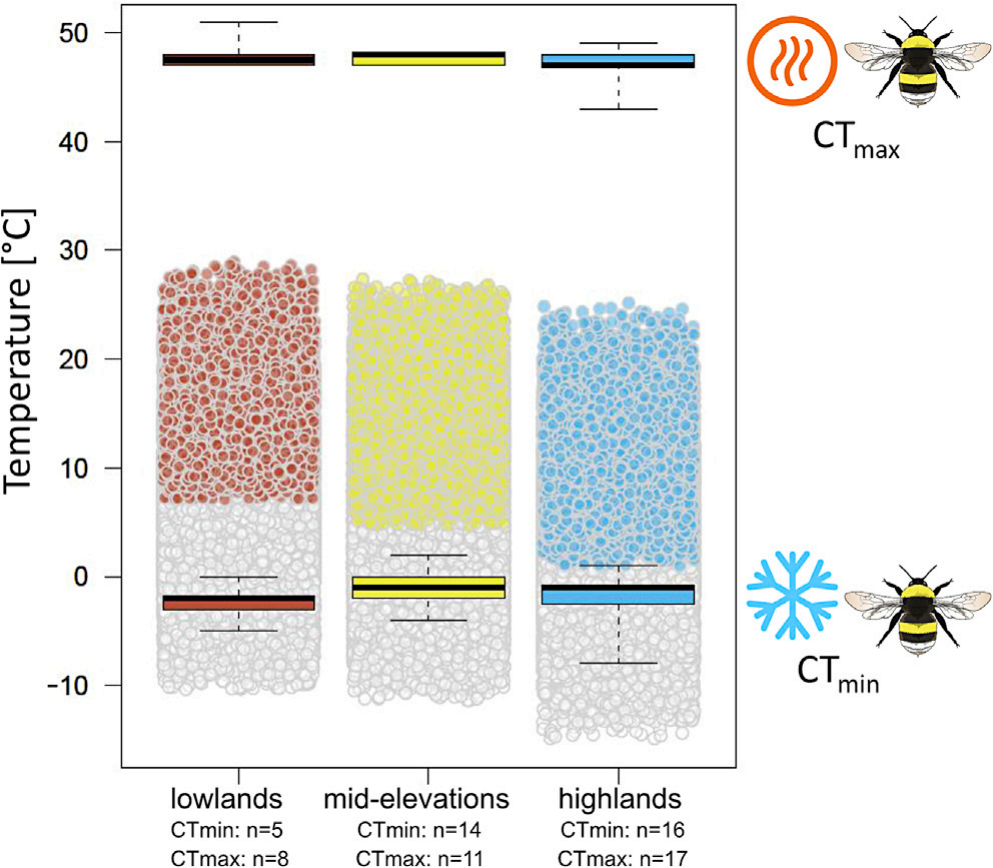
Thermal tolerance of *B. lucorum* along the elevation gradient Maximal (CTmax, upper boxplots) and minimal (Ctmin, lower boxplots) thermal tolerances for bumblebees at different elevational belts (714–1,017 m a.s.l.: lowland (red); 1,140–1,448 m a.s.l.: mid-elevation (yellow); 1,579–2,032 m a.s.l.: highland (blue). Dots in the middle represent hourly-measured temperature data in the study year in the respective elevational belts, with dots in color indicating summer temperatures measured from June to August, when the experiment was conducted. There were no significant differences in the mean CTmax and CTmin among elevational belts (ANOVA, *p* > 0.05). Boxes range from the first to the third quartile, whereas bold lines mark medians. Upper and lower whiskers mark the position of outliers.

We observed no difference in *B. lucorum* physiological thermal tolerance (mean CTmin and CTmax), when testing individuals from different elevational belts against each other (Figures 5 and Table S3) (ANOVA, CTmin: df = 2, *F* = 0.64, *p* = 0.532; CTmax: df = 2, *F* = 0.82, *p* = 0.45). Both CTmin and CTmax were neither affected by body size of the specimen, which we approached by measuring the intertegular distance (ITD) (CTmin: *F* = 0.60 *p* = 0.444; CTmax *F* = 0.21 *p* = 0.648) nor by dry body weight (CTmin: *F* = 0.42 *p* = 0.522; CTmax *F* = 0.33 *p* = 0.569).

### Effect of livestock grazing on bumblebee gene expression

**Figure 6:**
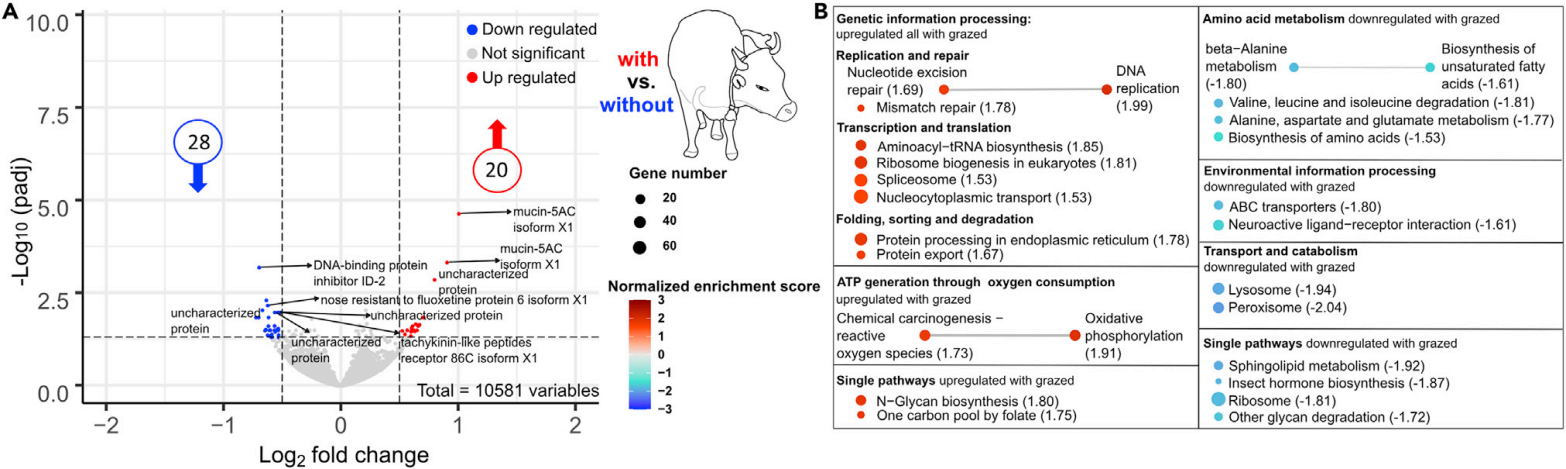
Effect of grazing on the gene expression of *B. lucorum* Summary of the RNA-seq results by Enrichment Map representation of the volcano plot (A) obtained from DESeq2 analysis (*x*-axis represents the log2 of the fold change, *y*-axis represents the negative decade logarithm of the significance value) and the ranked GSEA results (B) between *B. lucorum* samples originating from sites with and without livestock grazing. A detailed description of the different shown parameters can be obtained from Figures 2 and 3.

The GSEA analyses of changes in the different KEGG pathways showed an upregulation of genetic information processes (e.g. DNA replication, Aminoacyl-tRNA biosynthesis, Mismatch repair, Ribosome biogenesis in eukaryotes) from *B. lucorum* on sites with livestock grazing pressure (Figure 6B and Table S10). Additionally, oxygen transformation processes (reactive oxygen species, oxidative phosphorylation) are upregulated. On unmanaged grasslands, especially amino acid metabolism, environmental information processing, and transport/catabolism were upregulated in the transcriptome of *B. lucorum* (Figure 6B and Table S10). Besides the up- or downregulation of whole pathways, we observed 28 single downregulated genes in *B. lucorum* from sites with livestock grazing and 20 upregulated genes (Figure 6A and Table S9). Thirty percent of these genes were uncharacterized, whereas the remaining could not be assigned to a certain KEGG pathway.

## Discussion

### Transcriptional regulation of *B. lucorum* in different elevations

Along the elevational gradient in the Berchtesgaden National Park, where the mean annual temperature changes from 0 to 4.6°C, we identified several genes associated with elevation and thus also with temperature. However, candidate genes already associated with thermal stress in other studies did not show significant expression changes with the temperature changes studied in the experimental design of the elevational gradient (Liu et al., 2020a, 2020b; Pimsler et al., 2020). Some of those candidates, such as HSP are known to be responsive toward very different stressors such as heat, cold, osmotic, or oxidative stress, hypoxia, exposure to toxic substances, or infections as a protection mechanism for other proteins (Feder and Hofmann, 1999). Given that there was no regulation of HSP and other important stress-induced genes (Figures S1 and S2), we assume that *B. lucorum* is resilient against temperature changes in the range that occurs on the studied elevational gradient. Thermal stress was potentially not strong enough to trigger extra protection for protein functioning (Liu et al., 2020a). Finding no significant difference in the expression of candidate genes between elevational belts paralleled with the finding, that *B. lucorum* workers showed no elevational pattern in their physiological thermal tolerances (Figure 5). Again, temperature differences during the time of colony growth (May-August) are not reaching the critical thermal conditions to select for physiological adaptation or to trigger specific metabolic protection mechanisms. Alternatively, adaptive differences in critical minimum thermal limits, as detected in climate-chamber raised workers (Pimsler et al., 2020), might also have remained undetected in our study, because the foraging workers, which we collected in the field, may stem from colonies that are located at much lower or higher elevations; the collected workers may therefore be acclimatized to other environmental temperatures than those of the sampling locality.

Even though single candidate genes showed no significant up- or downregulation between elevational belts we detected a significant upregulation of pathways involved in energy generation with increasing elevation (Figure 3). Especially the carbon metabolism was upregulated in mid-elevation and highlands compared with lowland bumblebees. It is known that bumblebees utilize carbohydrates to power their flight; thus, these insects support flight by using sugars from plant nectar rather than using the fat body (Suarez et al., 2005). Additionally, besides the carbohydrate metabolism, we observed an upregulation in ATP generation through oxidative phosphorylation with higher elevation. Oxidative phosphorylation is known to generate cellular ATP in the mitochondrion of eukaryotic cells (Tripoli et al., 2005). It was shown that it can provide up to 70–95% of the ATP necessary for eukaryotic cells (Li et al., 2017; Mitterboeck et al., 2017). In contrast to a recent study (Masson et al., 2017) we showed that cold adaption in nature is not only supported by ATP production through oxidative phosphorylation, but by a combination with the carbohydrate metabolism. *Bombus lucorum* is responding to temperature and elevation changes through increased ATP production like bumblebee species from the high-elevation Tibet Plateau (Liu et al., 2020a, 2020b). This plastic “energy boost” might allow *B. lucorum* to occupy a large geographic range with different temperature regimes. To verify whether gene expression differences detected between elevational belts were related to temperature differences, we checked whether we find the same transcriptome response, when comparing individuals collected during different sampling temperatures, but within the same elevational belt (lowlands). Indeed, also in this subset, we could detect an enrichment in the carbohydrate ATP production pathways under cool sampling temperature. The differences in pathway regulation between sampling temperature categories were less statistically supported than the differences between elevational belts. Nevertheless, they were clear enough that the response to elevation was mainly driven by temperature and not by, for example, oxygen partial gas pressure differences between low and high elevation. Also, in the single significantly upregulated genes we could find an increase in 11 genes that are related to the carbon metabolism. Other regulated genes showed no clear pattern in a specific directed change in metabolic or functional pathways. Under the assumption that collected specimens originate from colonies nesting at lower altitudes, this suggests that energy-intensive long-distance flights from lower to higher altitudes alone do not trigger the observed transcriptional responses. How a frequent activation of “energetic boosters” impact colony fitness and growth, remains little understood. However, data from another study from that area suggests that bumblebee abundance increased in mid- and high elevations (but not in low elevations), when comparing a warmer year (2019) with a cooler year (2009) (Figure S3 and Maihoff et al., under revision). This may indicate, with the limitation of only two sampling years, that relative environmental stress-resistant bumblebees such as *B. lucorum* may initially profit from warming temperatures at higher elevations, as foraging flights become more energy-efficient and, therefore, more energy can be invested in colony growth.

### Livestock grazing triggers transcriptome response in *B. lucorum*

Here we show, that even extensive livestock grazing, as conducted in the National Park Berchtesgaden, triggers a transcriptome response in the bumblebee *B. lucorum.* These responses are detected despite the limitation that we do not know whether captured, foraging bumblebees also nested on the respective grazed sites. The majority of pathways upregulated under the grazing treatment were involved in genetic information processes like transcription, translation, replication, and repair of DNA (Figure 6). This suggests that a certain, yet unknown interaction with livestock is forcing bumblebees to express mechanisms to cope with damage or interference in their genome. Especially the mismatch repair system, which is conserved through the complete Tree of Life, is important for organisms to deal with DNA damage processes occurring from either environmental factors or metabolic processes inside the cell (Lee et al., 2016; Li, 2008). First, interactions with cattle dung itself, containing nitrogen and sulfur compounds, could be potentially harmful to the ground-nesting bumblebees and trigger the observed responses (Jensen, 2003). It is known that the accumulation of NOx up to a certain threshold can have a toxic effect, mainly through an increase in methaemoglobin, a hemoglobin derivative that cannot bind oxygen but prevents hemoglobin from releasing its oxygen, ultimately leading to hypoxia (Bachmann, 1937; Jensen, 2003; Lefevre et al., 2011). Data from the same study sites shows that the introduction of cattle is significantly increasing the concentration of nitrite and nitrate (NOx) in the soil (with livestock grazing: ∼29 mg NOx/kg soil; unmanaged: ∼6 mg NOx/kg soil; Table S11 and Figure S4). The toxicity of NOx to insects has so far been studied mainly in freshwater ecosystems and has received little attention in terrestrial systems (Camargo and Alonso, 2006; Eytcheson and Leblanc, 2018; Soucek and Dickinson, 2012; Tesfai et al., 1977; Throop and Lerdau, 2004).

From the composition of the pathways that are regulated, we would exclude a direct effect, like soil compaction damaging potential or existing ground nesting sites (Murray et al., 2012) of cattle activity on the sampled bumblebees, as this would probably not trigger a response in genetic information processes.

Alternatively, grazing might change the composition of plants, with plants being potentially toxic to bumblebees becoming more dominant on pastures, either due to a higher nitrogen input from NOx compounds or due to the selective consumption of other plants by cattle (Augustine and McNaughton, 1998; Carvell, 2002; Roulston and Goodell, 2011). It is known that several plants may contain certain secondary compounds (specific alkaloids or steroids) that are also present in their nectar or pollen and thus potentially toxic to bumblebees except the evolutionary specialized species- (Adler, 2000; Trunz et al., 2020; Villalona et al., 2020), or at least triggering the observed transcriptome responses (Xu et al., 2013). Interestingly, the presence of fertilizer itself has been shown to alter the chemical composition of nectar and pollen (ratio of essential amino acids is changing, and lower glucose and fructose levels, respectively) in the plant species *Succisa pratensis* (Family: Caprifoliaceae), which is also present in our study region, leading to higher mortality rates in the larvae of *B. terrestris* and less living workers (Ceulemans et al., 2017). We observe a downregulation in pathways that are involved in amino acid metabolisms, which could hint into the direction that grazing indeed is either changing the composition of amino acids of the available plants either directly or indirectly. However, to really show a causality between these effects, would require knowledge about which resources were actually used by the foraging bumblebees and the respective amino acid and sugar content of the flowers.

To date, there are no transcriptomic analyses of plant pollen or nectar toxins on the transcriptome of bumblebees or other bees, which makes a comparison with our dataset difficult. A comparison of our study with transcriptome work on the influence of insecticides revealed no overlap in the regulation of genetic processes; only oxidative stress seems to be a common trigger for the bees’ response to different kinds of stressors (Fent et al., 2020; Gao et al., 2020; Shi et al., 2017).

### Conclusion

In this study, we analyzed the transcriptome of *B. lucorum* workers, sampled along an elevational gradient from differently managed grassland sites in and around the National Park Berchtesgaden. We conclude that potential temperature constraints may be compensated by higher metabolic energy productions in our study system rather than by causing a transcriptional thermal stress response. Livestock Grazing, in contrast, led to an unexpected upregulation of processes involved in stress response, immunoregulation, and DNA-regeneration/repair. Although the underlying mechanisms remain partly enigmatic, our study demonstrates the complexity and resilience of the molecular response of natural pollinators to drivers of global change. As under expected global change, *B. lucorum* will most likely face land use change and temperature increase we conclude that rising temperature facilitates *B. lucorum* to colonize higher elevations, whereas fitness implications of even minor intensification of land use practices should raise our concern. A more comprehensive study, ideally of different species, in which whole colonies, with similar conditions, are monitored for several weeks, is needed to uncover the variables that lead to the effects we observed and to determine whether these changes ultimately affect the fitness of bumblebees in these regions.

### Limitations of the study

In this study, we provide an extensive transcriptomic dataset of *B. lucorum*, a widespread bumblebee species, which is due to its cold tolerance also common in alpine regions.

The bumblebees used in this study were caught while foraging at different study sites along the elevational gradient. Whereas this method has the advantage of capturing bumblebees in their natural environment, i.e. with all realistic abiotic and biotic interactions, it has the disadvantage that we have neither information on the age of individuals or colony identity, nor on the location of the nesting site. Vertical flights or age differences could therefore have masked existing patterns in the transcriptome response. Also, we are unable to link transcriptomic changes to the fitness of bumblebee colonies.

Another limitation is that we have only analyzed the data of one bumblebee species. Therefore, we cannot know whether the reported transcriptome responses to temperature and grazing, are general responses of bumblebee species in temperate alpine communities. In the next approach, it would be interesting to include more bumblebee species, also those with e.g. narrower elevational ranges.

## STAR★Methods

### Key resources table

**Table:** 

REAGENT or RESOURCE	SOURCE	IDENTIFIER
**Biological samples**		

In total 81 *Bombus lucorum* workers for transcriptomic analyse	Foraging in the National Park Berchtesgaden and its vicinity, Germany in 2019 (47,10° N, 12,15° E)	Identification by AIM - Advanced Identification Methods, Leipzig, Germany
In total 71 *Bombus lucorum* workers for thermal tolerance analyse	Foraging in the National Park Berchtesgaden and its vicinity, Germany in 2020 (47,10° N, 12,15° E)	NA

**Deposited data**		

Raw and analyzed RNA-seq data	This paper	GEO: GSE198931
Raw sequence fasta file for bumblebee identification	This paper	NCBI: SUB11540607
Supplementary Tables	This paper	Zenodo Data: https://doi.org/10.5281/zenodo.7077851
Bumblebee abundance data	This paper	https://doi.org/10.5061/dryad.80gb5mkt1

**Chemicals, peptides, and recombinant proteins**		

RNeasy Mini Kit	Qiagen, Hilden, Germany	https://www.qiagen.com
RNA 6000 Nano		
Kit Guide	Agilent, Santa Clara, United States	https://www.agilent.com
TruSeq Stranded mRNA Library Preparation Kit	Illumina, San Diego, United States	https://www.illumina.com

**Software and algorithms**		

R 4.1.2	The R Project for Statistical Computing (R Development Core Team, 2008)	https://www.r-project.org
Cutadapt (v2.5)	(Martin, 2011)	https://cutadapt.readthedocs.io/en/stable/
STAR (v2.7.2b)	(Dobin et al., 2013)	https://github.com/alexdobin/STAR
featureCounts (v1.6.4)	(Liao et al., 2014)	http://subread.sourceforge.net/
DESeq2 (v1.24.0)	(Love et al., 2014)	http://bioconductor.org/packages/release/bioc/html/DESeq2.html
ClusterProfiler (v3.12.0)	(Yu et al., 2012)	https://bioconductor.org/packages/release/bioc/html/clusterProfiler.html
EnhancedVolcano	(Blighe et al., 2019)	https://bioconductor.org/packages/release/bioc/html/EnhancedVolcano.html

**Other**		

FastPrep beat-beating system Savant FastPrep FP120-230	Thermo Fisher Scientific, Dreieich, Germany	https://www.thermofisher.com
2100 Bioanalyzer	Agilent, Santa Clara, United States	https://www.agilent.com
NextSeq 500 platform	Illumina, San Diego, United States	https://www.illumina.com
Eppendorf ThermoStat™ thermocycler	Eppendorf, Hamburg, Germany	https://www.eppendorf.com

### Resource availability

#### Lead contact

Further information and requests for resources and reagents should be directed to and will be fulfilled by the lead contact: Dr. Kristof Brenzinger (kristof.brenzinger@uni-wuerzburg.de).

#### Materials availability

This study did not generate new unique reagents.

### Experimental model and subject details

#### Study sites and bumblebee sampling

The study was conducted within the National Park Berchtesgaden and its vicinity (47,10° N, 12,15° E). The National Park is located within the limestone Alps in southern east Germany a region characterized by coniferous forest, alpine meadows, and mountain pastures. The later are grazed extensively by either cattle or sheep, used for hay production, or abandoned within the last 150 years. We collected bumblebees on 27 study sites in three elevational levels: lowlands (10 study sites from 641–1017 m a.s.l.), mid-elevations (9 study sites from 1105–1448 m a.s.l.), highlands (8 study sites from 1579–2032 m a.s.l.) (Figure 1A), corresponding to a temperature gradient from 9 to 4.6°C from lowland to highland study sites. Half of the study sites were used actively as pastures during the sampling season and in previous years; the other half comprised grasslands without livestock grazing (Hoiss et al., 2012). Study sites with and without livestock grazing were equally distributed along the climatic gradient such that both factors were uncorrelated and could be independently analysed.

#### Bumblebee collection and species identification for transcriptome analyses

In total, 81 individuals (Lowlands: 30; mid-elevation: 25; highlands: 26; Table S1) of *B. lucorum* were collected via sweep nets on 16 different study sites (lowlands: 6 study sites from 641–987 m a.s.l.; mid-elevations: 5 study sites from 1140–1420 m a.s.l; highlands: 5 study sites from 1579–1930 m a.s.l.) between end of July and mid of August 2019 between 9 a.m. and 7 p.m. We collected 5 workers per study site except for the highest site, where on one site less individuals were found, therefore on another site more individuals were captured. Immediately after catching, specimens were killed by severing the head from the thorax with sterilized scissors (flaming with 95% ethanol). Legs, wings, and the gut were removed with sterilized forceps before thorax and abdomen were transferred to DNA/RNA shield (Zymo Research, Eching, Germany), and roughly cut to allow shield absorption. The dissection procedure was conducted within 1 min to minimize the confounding effects of preparation on gene expression status. Samples were immediately stored on ice in the field. Legs were also placed on ice in separate vials for later species identification. After field trips, all samples were continuously stored at −20°C in a freezer until further processing. Species identification of bumblebees were confirmed by DNA-barcoding. Legs of all bumblebees were isolated and subsequently analysed via DNA-barcoding based on the cytochrome oxidase CO1 gene (Hebert et al., 2003, 2004) (AIM - Advanced Identification Methods, Leipzig, Germany) and annotated through BLAST and BOLD (The Barcode of Life Data System) databases (Ratnasingham and Hebert, 2007). All samples were accurately assigned to *B. lucorum* with >98.8% reference gene matching (Table S1). All sequence fasta files are stored at NCBI with the submission number SUB11540607. Unfortunately, we could not identify any clusters of specimens based on COI that could indicate colony affiliation or degree of relationship. Therefore, our study is limited in that we consider free-flying individuals with unknown relationships. Although it cannot be ruled out that individuals caught on a site belong to the same colony, we are confident that our design (several geographically distant sites in one altitude category or temperature category, see Figure 1) compensates for possible colony effects.

#### Bumblebee collection for the assessment of thermal tolerances

In total, 71 workers of *B. lucorum* (Table S3) were collected on 20 study sites (lowlands: 5 study sites from 714–1017 m a.s.l.; mid-elevations: 8 study sites from 1140–1448 m a.s.l; highlands: 7 study sites from 1579–2032 m a.s.l.) between June and August 2020, the study sites included the same 9 out of 16 sites used for collecting bumblebees for transcriptome analyses and used 11 additional sites (Figure 1C). We are much aware of the fact that works of *B. lucorum* cannot be reliably differentiated from *B. terrestris, B. cryptarum and B.magnus* by morphological characters alone (Bossert et al., 2016; Williams et al., 2012). Unfortunately, financial constraints did not allow us to barcode the respective samples. However, since out of 117 randomly collected individuals in the study region (81 were used for transcriptomic data) we could identify only one male of *B. terrestris* and 116 females of *B. lucorum* by DNA barcoding (see above), we conclude that our study region is vastly dominated by *B. lucorum*.

### Method details

#### Bumblebee abundance comparing a warmer and a hotter year (2009 vs. 2019)

The bumblebee abundance data, as well as the data of this paper, were generated within the Adapt project ("Adaptation of alpine pollinators in Times of global change") but will be published by F.M. embedded in a larger dataset soon. Upon acceptance of this manuscript raw data will be available at https://doi.org/10.5061/dryad.80gb5mkt1.

In addition to the 27 study sites used in this study, wild bees were observed in a standardized manner on a further 6 sites over the entire season (May to September) in 2009 and 2019. Details of the sampling method can be found in Hoiss et al. (2012), which published extended data of the sampling conducted in 2009. While the mean annual temperature on the selected sites was in 2019 0.9°C warmer than 2009. The temperature in the region has also increased over the 10-year period.

#### RNA extraction and sequencing

RNA was extracted from whole bumblebee bodies without the gut (RNeasy Mini Kit, Qiagen, Hilden, Germany), because of the role of musculature for thermogenesis/thermal tolerance (Andersen and Overgaard, 2019) and the importance of homogenous tissues in RNAseq (Johnson et al., 2013). Thorax and abdomen were disrupted in a FastPrep bead beating system (Savant FastPrep FP120-230, Thermo Fisher Scientific, Dreieich, Germany). Afterwards, the RNA was extracted in accordance with manufacturer’s protocol.

RNA quality was checked using a 2100 Bioanalyzer with the RNA 6000 Nano kit (Agilent, Santa Clara, United States). The RIN for the samples was from 2,7 – 9,0. DNA libraries suitable for sequencing were prepared from 400 ng of total RNA with oligo-dT capture beads for poly-A-mRNA enrichment using the TruSeq Stranded mRNA Library Preparation Kit (Illumina, San Diego, United States) according to manufacturer’s instructions (½ volume, 4 samples: full volume). After 15 cycles of PCR amplification, the size distribution of the barcoded DNA libraries was estimated ∼285 bp by electrophoresis on Agilent DNA 1000 Bioanalyzer microfluidic chips. Sequencing of pooled libraries, spiked with 1% PhiX control library, was performed at 22–42 million reads/sample in single-end mode with 75 nt read length on the NextSeq 500 platform (Illumina) using 5 High Output sequencing kits.

#### Processing of RNA-seq data

Demultiplexed FASTQ files were generated with bcl2fastq2 v2.20.0.422 (Illumina). To assure high sequence quality, Illumina reads were quality- and adapter-trimmed via Cutadapt (Martin, 2011) version 2.5 using a cutoff Phred score of 20 in NextSeq mode, and reads without any remaining bases were discarded (command line parameters: --nextseq-trim = 20 -m 1 -a AGATCGGAAGAGCACACGTCTGAACTCCAGTCAC). Processed reads were subsequently mapped to the bumblebee genome (GCF_000214255.1/Bter_1.0 primary assembly) using STAR v2.7.2b with default parameters based on RefSeq annotation release 102 for Bter_1.0 (Dobin et al., 2013). Read counts on exon level summarized for each gene with gene_biotype protein_coding were generated using featureCounts v1.6.4 from the Subread package (Liao et al., 2014). Multi-mapping and multi-overlapping reads were counted strand-specific and reversely stranded with a fractional count for each alignment and overlapping feature (command line parameters: -s 2 -t exon M O -fraction). The raw data is stored at GEO: GSE198931.

### Quantification and statistical analysis

RNA-seq read counts were utilized to identify differentially expressed genes using DESeq2 (Love et al., 2014) version 1.24.0. Read counts were normalized by DESeq2 and fold-change shrinkage was applied by setting the parameter “betaPrior = TRUE.” For pairwise comparison between groups, three independent analyses were conducted based on different sample sets and group assignments. At first, all *B. lucorum* samples were assigned to three categories along the elevation gradient (lowland, mid-elevation, highland). Next, to check for a temperature effect independent of an elevation effect (like changes in oxygen pressure, radiation, or vegetation), an additional analysis was conducted using only the lowland samples, which were subdivided into two groups based on temperature during sampling (high T: 20.6 – 23.5°C vs. low T: 14.8–18.5°C; Table S2). For these analyses, we could only use the samples from the lowlands and not from higher elevational belts, as those were the only samples which we could group into two similarly sample-sized groups that could be sufficiently distinguished by temperature. For a third analysis, all samples were alternatively grouped based on collection at grazing and non-grazing sites (Table S1). Differential expression of genes was assumed at an adjusted p-value (padj) after Benjamini-Hochberg correction < 0.05 and |log2FoldChange| ≥ 0.5. clusterProfiler (Yu et al., 2012) version 3.12.0 was used to perform functional enrichment analyses based on Kyoto Encyclopedia of Genes and Genomes (KEGG) pathways. Here, the GSEA function (Subramanian et al., 2005) was applied for gene set enrichment analysis considering the DESeq2 log2FoldChange of all analysed genes and the function emapplot was used for result visualization (Reimand et al., 2019). This method was used in other recently studies on bee transcriptomes (Chen et al., 2021; Holman et al., 2019; Huang et al., 2021).

#### Measurement of thermal tolerance of *B. lucorum*

To measure the thermal tolerance limits of *B. lucorum*, specimens were transported in 1.5 mL, ventilated plastic tubes to the laboratory (field station) or laboratory substitute (mountain hut). Each individual was offered cotton wool soaked with sugar water during the time of transport, but at least ten minutes immediately before the start of the experiment to avoid an influence of the hunger state on thermal performance. The interest in the sugar solution always decreased before the 10 minutes had elapsed (indicated by animals’ movement away from the sugar source in the transport vial). Individuals showing injuries or strong signs of lethargy from transport after the feeding, were removed from the experiment.

To access maximal thermal tolerance (CTmax) and minimal thermal tolerance (CTmin), we recorded the irreversible thermal tolerance limit in each treatment, i.e. the minimal and maximal temperature at which postural control was lost (Hamblin et al., 2017) and controlled mobility (i.e. a posture that allows normal walking or sitting) cannot be reactivated anymore by stimuli (= loss of the so called “righting response”) (Kellermann et al., 2012; Oyen et al., 2016). Note that the loss of postural control also includes described behaviours such as motor spasticity and body curling (Oyen and Dillon, 2018). These extreme behaviours were not distinguished here from motionless behaviours as it excludes an ecologically relevant behaviour (foraging/flying).

In the assessment, we exposed single bees within 2 mL (small bees) or 5 mL (large bees) plastic vials to decreasing or respectably increasing temperatures generated within a programmable thermocycler (Eppendorf ThermoStatC, Hamburg, Germany) (Diamond et al., 2012; Peters et al., 2016). To ensure free exchange of gas during the cause of the experiment, vials were closed with fresh cotton balls and placed into the thermocycler. We programmed two temperature patterns that guaranteed a stepwise time-regulated temperature drop or temperature rise. To identify CTmin (n = 35), specimens were held ten minutes at a common initial temperature of 14°C. Then temperature was two times reduced by 2°C (up to 10°C), and from then in 1°C intervals. For the assessment of CTmax (n = 36), specimens were kept ten minutes at a common initial temperature of 20°C. Temperature was first increased by 2°C to 24°C, followed by 1°C intervals. This approach with first 2°C and then 1°C steps is since in preliminary studies all individuals tolerated 10°C or 26°C and thus we could shorten the total duration of the experiment. In both treatments, each temperature was held for five minutes; time counting started when the set temperature was reached. After each 5 minutes at the given temperature gently flicking the vial was used as a stimuli to induce a righting response (Kellermann et al., 2012; Peters et al., 2016). Our approach to determine the CTmin, first applied to multiple taxa (Diamond et al., 2012), is also suitable for bumblebees (Peters et al., 2016). However it comes along with some limitations: As bumblebees have thermogenic capabilities, the temperature at which we observed the loss of postural control may not correspond to thoracic temperatures of bee (Oyen and Dillon, 2018), why our measurements are not directly comparable with more precise measurements of CTmin. Further righting response are discussed to be influenced by individuals’ motivation and hence causes variation which not necessary reflects pure physiological thresholds (Lutterschmidt and Hutchison, 1997; Oyen and Dillon, 2018). Compared to other methods with a continuous temperature increase, our stepwise temperature increases with 5 min hold time is rather coarsely resolved. Also, the rate of temperature increase per minute is reduced, which can lead to higher CTmin and lower CTmax values (Oyen and Dillon, 2018). Nevertheless, our approach should be precise enough to capture intraspecific variation in thermal tolerance.

As variation in thermal tolerances can be influenced by body size (Peters et al., 2016) and vial size during measurement (Oyen and Dillon, 2018), we assessed body size by measuring the intertegular distance and body dry weight of each individual (Cane, 1987) and tested all three variables (body size, weight, vial size) using a linear model (lm: ∼ body size+ weight+ vial size) and subsequent ANOVA type II. Neither vial size during experiment had no significant an effect on bee thermal tolerances (CTmin: *F* = 0.01 *p* = 0.981; CTmax *F* = 0.75 *p* = 0.392). We tested by analysis of variance (ANOVA, typ I for balanced data) whether the thermal tolerance (mean CTmin and CTmax) differed between the elevational belts. These analyses were done using R version 4.1.2 (Core TEAM, 2020).

#### Analyses of NOx composition along the elevation gradient

Soil samples were collected in summer 2020 from similar sites where the bumblebees were catch. Soil was non-destructively sampled using a soil core (diameter x height: 5 × 15 cm) in a random sampling approach, in total 5 replicates were taken per site. Nutrient concentrations in the soil samples (NOx-) were determined in 1 M KCl (1:5 dilution) extract using a SEAL QuAAtro SFA autoanalyzer (Beun-de Ronde B.V. Abcoude, the Netherlands) at the Netherland Institute of Ecology (Wageningen, the Netherlands). Data is stored in the Table S10.

#### Temperature extraction

Mean annual temperature (MAT) of the sampling year (2019) for each study site and temperature during sampling (mean temperature one hour before and after the specimen was caught) was predicted from temperature data extracted from 17 adjacent climate stations. The respective climate stations were located in and around the national park, covering an elevational range from 653 to 2645 m a.s.l. Climate stations recorded temperature in 10-min intervals, which were first averaged per day (MAT) or hour (temperature during sampling) respectively. For the prediction of MAT, we then fitted generalized additive models (GAMs) with a smooth term of elevation, day length in minutes (as a proxy for season), and date (to account for daily weather conditions on respective days). Predicted daily temperatures were then averaged per year for each study site. For the prediction of temperature during sampling, we fitted GAMs with a smooth term of elevation and day lengths in minutes (as a proxy for season), and a combined factor for date and hour (e.g. 2019-01-01_hour10, to account for the hourly weather conditions on the respective day). Precise elevations of climate stations and study site centres were extracted from a digital elevational model with 1-m resolution.

## Data Availability

• RNA-seq data have been deposited at NCBI and are publicly available as of the date of publication (GEO: GSE198931). Accession numbers are listed in the key resources table. Raw sequence fasta file for bumblebee identification have been deposited at NCBI and are publicly available as of the date of publication (SUB11540607). Additional data, metadatafiles and in general all Supplementary tables are deposited at https://doi.org/10.5281/zenodo.7077851. • This paper does not report original code. • Any additional information required to reanalyse the data reported in this paper is available from the lead contact upon request.

## Supplemental information

Document S1. Figures S1–S4

Table S1. Metadatafile and overview of all B. lucorum samples used for the transcriptomic study, related to Figure 1A

Table S2. Metadatafile and overview of all Bombus lucorum samples from lowlands used for the analyses of differences occurring between temperatures during sampling, related to Figure 4

Table S3. Metadatafile, overview and results of all B. lucorum samples used for the analyses of the thermal tolerance measurement, related to Figure 1C

Table S4. Overview of all sequenced transcripts DESeq2 raw count table for all single samples and the groupwise comparison of elevation, lowland temperature, and grazed site, related to RNA-seq analyses

Table S5. Overview of differently significant regulated genes between the different elevation belts, related to Figure 2

Table S6. Overview of differently significant regulated KEGG pathways between the different elevation belts, related to Figure 3

Table S7. Overview of differently significant regulated genes under low and high sampling temperature during sampling in lowland, related to Figure 4A

Table S8. Overview of differently significant regulated KEGG pathways under low and high sampling temperature during sampling in lowland, related to Figure 4B

Table S9. Overview of differently significant regulated genes under livestock grazing vs without livestock, related to Figure 6A

Table S10. Overview of differently significant regulated KEGG pathways under livestock grazing vs without livestock, related to Figure 6B

Table S11. Overview of all soil samples used for measuring NOx on sites with livestock grazing and without, related to Figure S3

## References

[bib1] Adler L.S. (2000). The ecological significance of toxic nectar. Oikos.

[bib2] Andersen M.K., Overgaard J. (2019). The central nervous system and muscular system play different roles for chill coma onset and recovery in insects. Comp. Biochem. Physiol. Mol. Integr. Physiol..

[bib3] Augustine D.J., McNaughton S.J. (1998). Ungulate effects on the functional species composition of plant communities : herbivore selectivity and plant tolerance. J. Wildl. Manage..

[bib4] Bachmann W.E. (1937). The action of nitrite on haemoglobin in the absence of oxygen. Proc. R. Soc. Lond. Ser. B Biol. Sci..

[bib5] Biella P., Bogliani G., Cornalba M., Manino A., Neumayer J., Porporato M., Rasmont P., Milanesi P. (2017). Distribution patterns of the cold adapted bumblebee Bombus alpinus in the Alps and hints of an uphill shift (Insecta: Hymenoptera: Apidae). J. Insect Conserv..

[bib6] Blasco-Lavilla N., García-Reina A., De la Rúa P. (2021). Mild thermal stress does not negatively affect immune gene expression in the bumblebee Bombus terrestris. Apidologie.

[bib7] Blighe K., Rana S., Turkes E., Ostendorf B., Lewis M. (2019). https://github.com/kevinblighe/EnhancedVolcano.

[bib8] Bossert S., Gereben-Krenn B.-A., Neumayer J., Schneller B., Krenn H.W. (2016). The cryptic Bombus lucorum complex (Hymenoptera: Apidae) in Austria: phylogeny, distribution, habitat usage and a climatic characterization based on COI sequence data. Zool. Stud..

[bib9] Camargo J.A., Alonso Á. (2006). Ecological and toxicological effects of inorganic nitrogen pollution in aquatic ecosystems: a global assessment. Environ. Int..

[bib10] Cane J.H. (1987). Estimation of bee size using intertegular span ( Apoidea ). J. Kans. Entomol. Soc..

[bib11] Carvell C. (2002). Habitat use and conservation of bumblebees (Bombus spp.) under different grassland management regimes. Biol. Conserv..

[bib12] Ceulemans T., Hulsmans E., Vanden Ende W., Honnay O. (2017). Nutrient enrichment is associated with altered nectar and pollen chemical composition in Succisa pratensis Moench and increased larval mortality of its pollinator Bombus terrestris L. PLoS One.

[bib13] Chen H., Wu G., Zhou H., Dai X., Steeghs N.W.F., Dong X., Zheng L., Zhai Y. (2021). Hormonal regulation of reproductive diapause that occurs in the year-round mass rearing of Bombus terrestris queens. J. Proteome Res..

[bib14] Corbet S.A., Fussell M., Ake R., Fraser A., Gunson C., Savage A., Smith K. (1993). Temperature and the pollinating activity of social bees. Ecol. Entomol..

[bib15] Diamond S.E., Nichols L.M., McCoy N., Hirsch C., Pelini S.L., Sanders N.J., Ellison A.M., Gotelli N.J., Dunn R.R. (2012). A physiological trait-based approach to predicting the responses of species to experimental climate warming. Ecology.

[bib16] Dobin A., Davis C.A., Schlesinger F., Drenkow J., Zaleski C., Jha S., Batut P., Chaisson M., Gingeras T.R. (2013). STAR: ultrafast universal RNA-seq aligner. Bioinformatics.

[bib17] Dunning L.T., Dennis A.B., Sinclair B.J., Newcomb R.D., Buckley T.R. (2014). Divergent transcriptional responses to low temperature among populations of alpine and lowland species of New Zealand stick insects (Micrarchus). Mol. Ecol..

[bib18] Dzialowski E.M., Tattersall G.J., Nicol S.C., Frappell P.B. (2014). Fluctuations in oxygen influence facultative endothermy in Bumblebees. J. Exp. Biol..

[bib19] Eytcheson S.A., Leblanc G.A. (2018). Hemoglobin levels modulate nitrite toxicity to Daphnia magna. Sci. Rep..

[bib20] Feder M.E., Hofmann G.E. (1999). Heat-shock proteins, molecular chaperones, and the stress response: evolutionary and ecological physiology. Annu. Rev. Physiol..

[bib21] Fent K., Schmid M., Christen V. (2020). Global transcriptome analysis reveals relevant effects at environmental concentrations of cypermethrin in honey bees (Apis mellifera). Environ. Pollut..

[bib22] Fourcade Y., Åström S., Öckinger E. (2019). Climate and land-cover change alter bumblebee species richness and community composition in subalpine areas. Biodivers. Conserv..

[bib23] Gao J., Jin S.S., He Y., Luo J.H., Xu C.Q., Wu Y.Y., Hou C.S., Wang Q., Diao Q.Y. (2020). Physiological analysis and transcriptome analysis of asian honey bee (Apis cerana cerana) in response to sublethal neonicotinoid imidacloprid. Insects.

[bib24] Geue J.C., Thomassen H.A. (2020). Unraveling the habitat preferences of two closely related bumble bee species in Eastern Europe. Ecol. Evol..

[bib25] Habel J.C., Samways M.J., Schmitt T. (2019). Mitigating the precipitous decline of terrestrial European insects: requirements for a new strategy. Biodivers. Conserv..

[bib26] Hallmann C.A., Sorg M., Jongejans E., Siepel H., Hofland N., Schwan H., Stenmans W., Müller A., Sumser H., Hörren T. (2017). More than 75 percent decline over 27 years in total flying insect biomass in protected areas. PLoS One.

[bib27] Halsch C.A., Shapiro A.M., Fordyce J.A., Nice C.C., Thorne J.H., Waetjen D.P., Forister M.L. (2021). Insects and recent climate change. Proc. Natl. Acad. Sci. USA.

[bib28] Hamblin A.L., Youngsteadt E., López-Uribe M.M., Frank S.D. (2017). Physiological thermal limits predict differential responses of bees to urban heat-island effects. Biol. Lett..

[bib29] Hebert P.D.N., Cywinska A., Ball S.L., Jeremy R. (2003). Biological identifications through DNA barcodes. Proc. Biol. Sci..

[bib30] Hebert P.D.N., Penton E.H., Burns J.M., Janzen D.H., Hallwachs W. (2004). Ten species in one: DNA barcoding reveals cryptic species in the neotropical skipper butterfly Astraptes fulgerator. Proc. Natl. Acad. Sci. USA.

[bib31] Heinrich B. (1979). Thermoregulation of African and European honeybees during foraging, attack, and hive exits and returns. J. Exp. Biol..

[bib32] Heinrich B. (1974). Thermoregulation in Endothermic Insects: body temperature is closely attuned to activity and energy supplies. Science.

[bib33] Heinrich B. (1972). Energetics of temperature regulation and foraging in a bumblebee, Bombus terricola kirby. J. Comp. Physiol..

[bib34] Heinrich B., Vogt F.D. (1993). Abdominal temperature regulation by arctic bumblebees. Physiol. Zool..

[bib35] Herbertsson L., Khalaf R., Johnson K., Bygebjerg R., Blomqvist S., Persson A.S. (2021). Long-term data shows increasing dominance of Bombus terrestris with climate warming. Basic Appl. Ecol..

[bib36] Hines H.M. (2008). Historical biogeography, divergence times, and diversification patterns of bumble bees (Hymenoptera: Apidae: Bombus). Syst. Biol..

[bib37] Hoffmann A.A., Willi Y. (2008). Detecting genetic responses to environmental change. Nat. Rev. Genet..

[bib38] Hoiss B., Krauss J., Potts S.G., Roberts S., Steffan-Dewenter I. (2012). Altitude acts as an environmental filter on phylogenetic composition, traits and diversity in bee communities. Proc. Biol. Sci..

[bib39] Holman L., Helanterä H., Trontti K., Mikheyev A.S. (2019). Comparative transcriptomics of social insect queen pheromones. Nat. Commun..

[bib40] Huang M., Dong J., Guo H., Xiao M., Wang D. (2021). Identification of long noncoding RNAs reveals the effects of dinotefuran on the brain in Apis mellifera (Hymenopptera: Apidae). BMC Genom..

[bib41] Jensen F.B. (2003). Nitrite disrupts multiple physiological functions in aquatic animals. Comp. Biochem. Physiol. Mol. Integr. Physiol..

[bib42] Johnson B.R., Atallah J., Plachetzki D.C. (2013). The importance of tissue specificity for RNA-seq: highlighting the errors of composite structure extractions. BMC Genom..

[bib43] Keller I., Alexander J.M., Holderegger R., Edwards P.J. (2013). Widespread phenotypic and genetic divergence along altitudinal gradients in animals. J. Evol. Biol..

[bib44] Kellermann V., Overgaard J., Hoffmann A.A., Fløjgaard C., Svenning J.C., Loeschcke V. (2012). Upper thermal limits of Drosophila are linked to species distributions and strongly constrained phylogenetically. Proc. Natl. Acad. Sci. USA.

[bib45] Körner C. (2007). The use of “altitude” in ecological research. Trends Ecol. Evol..

[bib46] Lee V., Murphy A., Le D.T., Diaz L.A. (2016). Mismatch repair deficiency and response to immune checkpoint blockade. Oncologist.

[bib47] Lefevre S., Jensen F.B., Huong D.T.T., Wang T., Phuong N.T., Bayley M. (2011). Effects of nitrite exposure on functional haemoglobin levels, bimodal respiration, and swimming performance in the facultative air-breathing fish Pangasianodon hypophthalmus. Aquat. Toxicol..

[bib48] Li G.M. (2008). Mechanisms and functions of DNA mismatch repair. Cell Res..

[bib49] Li Y., Zhang R., Liu S., Donath A., Peters R.S., Ware J., Misof B., Niehuis O., Pfrender M.E., Zhou X. (2017). The molecular evolutionary dynamics of oxidative phosphorylation (OXPHOS) genes in Hymenoptera. BMC Evol. Biol..

[bib50] Liao Y., Smyth G.K., Shi W. (2014). FeatureCounts: an efficient general purpose program for assigning sequence reads to genomic features. Bioinformatics.

[bib51] Liu Y., Jin H., Naeem M., An J. (2020). Comparative transcriptome analysis reveals regulatory genes involved in cold tolerance and hypoxic adaptation of high-altitude Tibetan bumblebees. Apidologie.

[bib52] Liu Y., Zhao H., Luo Q., Yang Y., Zhang G., Zhou Z., Naeem M., An J. (2020). De Novo transcriptomic and metabolomic analyses reveal the ecological adaptation of high-altitude bombus pyrosoma. Insects.

[bib53] Love M.I., Huber W., Anders S. (2014). Moderated estimation of fold change and dispersion for RNA-seq data with DESeq2. Genome Biol..

[bib54] Lutterschmidt W.I., Hutchison V.H. (1997). The critical thermal maximum: history and critique. Can. J. Zool..

[bib55] Maebe K., De Baets A., Vandamme P., Vereecken N.J., Michez D., Smagghe G. (2021). Impact of intraspecific variation on measurements of thermal tolerance in bumble bees. J. Therm. Biol..

[bib56] Maebe K., Hart A.F., Marshall L., Vandamme P., Vereecken N.J., Michez D., Smagghe G. (2021). Bumblebee resilience to climate change, through plastic and adaptive responses. Glob. Chang. Biol..

[bib57] Marshall H., Lonsdale Z.N., Mallon E.B. (2019). Methylation and gene expression differences between reproductive and sterile bumblebee workers. Evol. Lett..

[bib58] Marshall L., Perdijk F., Dendoncker N., Kunin W., Roberts S., Biesmeijer J.C. (2020). Bumblebees moving up: shifts in elevation ranges in the Pyrenees over 115 years. Proc. Biol. Sci..

[bib59] Martin M. (2011). Cutadapt removes adapter sequences from high-throughput sequencing reads. EMBnet. J..

[bib60] Martinet B., Dellicour S., Ghisbain G., Przybyla K., Zambra E., Lecocq T., Boustani M., Baghirov R., Michez D., Rasmont P. (2021). Global effects of extreme temperatures on wild bumblebees. Conserv. Biol..

[bib61] Masson S.W.C., Hedges C.P., Devaux J.B.L., James C.S., Hickey A.J.R. (2017). Mitochondrial glycerol 3-phosphate facilitates bumblebee pre-flight thermogenesis. Sci. Rep..

[bib62] Mayor J.R., Sanders N.J., Classen A.T., Bardgett R.D., Clément J.C., Fajardo A., Lavorel S., Sundqvist M.K., Bahn M., Chisholm C. (2017). Elevation alters ecosystem properties across temperate treelines globally. Nature.

[bib63] Michener C.D. (2007).

[bib64] Milchunas D.G., Sala O.E., Lauenroth W.K. (1988). A generalized model of the effects of grazing by large herbivores on grassland community structure. Am. Nat..

[bib65] Mitterboeck T.F., Liu S., Adamowicz S.J., Fu J., Zhang R., Song W., Meusemann K., Zhou X. (2017). Positive and relaxed selection associated with flight evolution and loss in insect transcriptomes. GigaScience.

[bib66] Moreira X., Castagneyrol B., Abdala-Roberts L., Traveset A. (2019). A meta-analysis of herbivore effects on plant attractiveness to pollinators. Ecology.

[bib67] Murray T.E., Fitzpatrick Ú., Byrne A., Fealy R., Brown M.J.F., Paxton R.J. (2012). Local-scale factors structure wild bee communities in protected areas. J. Appl. Ecol..

[bib68] Olff H., Ritchie M.E. (1998). Importance of herbivore type and scale. Trends Ecol. Evol..

[bib69] Oyen K.J., Dillon M.E. (2018). Critical thermal limits of bumblebees (Bombus impatiens) are marked by stereotypical behaviors and are unchanged by acclimation, age or feeding status. J. Exp. Biol..

[bib70] Oyen K.J., Giri S., Dillon M.E. (2016). Altitudinal variation in bumble bee (Bombus) critical thermal limits. J. Therm. Biol..

[bib71] Paaijmans K.P., Heinig R.L., Seliga R.A., Blanford J.I., Blanford S., Murdock C.C., Thomas M.B. (2013). Temperature variation makes ectotherms more sensitive to climate change. Glob. Chang. Biol..

[bib72] Pers-Kamczyc E., Tyrała-Wierucka Ż., Rabska M., Wrońska-Pilarek D., Kamczyc J. (2020). The higher availability of nutrients increases the production but decreases the quality of pollen grains in Juniperus communis L. J. Plant Physiol..

[bib73] Peters M.K., Peisker J., Steffan-Dewenter I., Hoiss B. (2016). Morphological traits are linked to the cold performance and distribution of bees along elevational gradients. J. Biogeogr..

[bib74] Pimsler M.L., Oyen K.J., Herndon J.D., Jackson J.M., Strange J.P., Dillon M.E., Lozier J.D. (2020). Biogeographic parallels in thermal tolerance and gene expression variation under temperature stress in a widespread bumble bee. Sci. Rep..

[bib75] Potts S.G., Biesmeijer J.C., Bommarco R., Breeze T.D., Carvalheiro L.G., Franzén M., González-Varo, Schweiger O. (2015).

[bib76] Pyke G.H., Thomson J.D., Inouye D.W., Miller T.J. (2016). Effects of climate change on phenologies and distributions of bumble bees and the plants they visit. Ecosphere.

[bib77] R Development Core Team (2008). http://www.R-project.org.

[bib78] Rasmont P., Franzén M., Lecocq T., Harpke A., Roberts S.P.M., Biesmeijer J.C., Castro L. (2015).

[bib79] Ratnasingham S., Hebert P.D.N. (2007). BOLD : the barcode of life data system. Mol. Ecol. Notes.

[bib80] Reimand J., Isserlin R., Voisin V., Kucera M., Tannus-Lopes C., Rostamianfar A., Wadi L., Meyer M., Wong J., Xu C. (2019). Pathway enrichment analysis and visualization of omics data using g:Profiler, GSEA, Cytoscape and EnrichmentMap. Nat. Protoc..

[bib81] Roulston T.H., Goodell K. (2011). The role of resources and risks in regulating wild bee populations. Annu. Rev. Entomol..

[bib82] Seibold S., Gossner M.M., Simons N.K., Blüthgen N., Müller J., Ambarlı D., Ammer C., Bauhus J., Fischer M., Habel J.C. (2019). Arthropod decline in grasslands and forests is associated with landscape-level drivers. Nature.

[bib83] Shi T.F., Wang Y.F., Liu F., Qi L., Yu L.S. (2017). Sublethal effects of the neonicotinoid insecticide thiamethoxam on the transcriptome of the honey bees (Hymenoptera: Apidae). J. Econ. Entomol..

[bib84] Sivan A., Shriram A.N., Muruganandam N., Thamizhmani R. (2017). Expression of heat shock proteins (HSPs) in Aedes aegypti (L) and Aedes albopictus (Skuse) (Diptera: Culicidae) larvae in response to thermal stress. Acta Trop..

[bib85] Sjödin N.E. (2007). Pollinator behavioural responses to grazing intensity. Biodivers. Conserv..

[bib86] Somero G.N. (2005). Linking biogeography to physiology: evolutionary and acclimatory adjustments of thermal limits. Front. Zool..

[bib87] Soucek D.J., Dickinson A. (2012). Acute toxicity of nitrate and nitrite to sensitive freshwater insects, mollusks, and a crustacean. Arch. Environ. Contam. Toxicol..

[bib88] Sponsler D., Requier F., Kallnik K., Classen A., Maihoff A.F., Sieger J., Steffan-Dewenter I. (2022). Contrasting patterns of richness, abundance, and turnover in mountain bumble bees and their floral hosts. Ecology.

[bib89] Suarez R.K., Darveau C.A., Welch K.C., O’Brien D.M., Roubik D.W., Hochachka P.W. (2005). Energy metabolism in orchid bee flight muscles: carbohydrate fuels all. J. Exp. Biol..

[bib90] Subramanian A., Tamayo P., Mootha V.K., Mukherjee S., Ebert B.L., Gillette M.A., Paulovich A., Pomeroy S.L., Golub T.R., Lander E.S., Mesirov J.P. (2005). Gene set enrichment analysis: a knowledge-based approach for interpreting genome-wide expression profiles. Proc. Natl. Acad. Sci. USA.

[bib91] Surholt B., Greive H., Baal T., Bertsch A. (1990). Non-shivering thermogenesis in asynchronous flight muscles of bumblebees? Comparative studies on males of Bombus terrestris, Xylocopa sulcatipes and Acherontia atropos. Comp. Biochem. Physiol. Physiol..

[bib92] Tesfai K., Mallik M.A.B., Pancholy S.K. (1977). Possible formation of a carcinogenic nitrosamine from an insecticide, phosphamidon, and nitrite in soil. Proc. Oklahoma Acad. Sci..

[bib93] Throop H.L., Lerdau M.T. (2004). Effects of nitrogen deposition on insect herbivory: implications for community and ecosystem processes. Ecosystems.

[bib94] Tripoli G., D’Elia D., Barsanti P., Caggese C. (2005). Comparison of the oxidative phosphorylation (OXPHOS) nuclear genes in the genomes of Drosophila melanogaster, Drosophila pseudoobscura and Anopheles gambiae. Genome Biol..

[bib95] Trunz V., Lucchetti M.A., Bénon D., Dorchin A., Desurmont G.A., Kast C., Rasmann S., Glauser G., Praz C.J. (2020). To bee or not to bee: the ‘raison d’être’ of toxic secondary compounds in the pollen of Boraginaceae. Funct. Ecol..

[bib96] Villalona E., Ezray B.D., Laveaga E., Agrawal A.A., Ali J.G., Hines H.M. (2020). The role of toxic nectar secondary compounds in driving differential bumble bee preferences for milkweed flowers. Oecologia.

[bib97] Vogt F.D. (1986). Thermoregulation in bumblebee colonies. I. Thermoregulatory versus. Physiol. Zool..

[bib98] Wagner D.L., Grames E.M., Forister M.L., Berenbaum M.R., Stopak D. (2021). Insect decline in the Anthropocene: death by a thousand cuts. Proc. Natl. Acad. Sci. USA.

[bib99] Williams P.H. (1998). An annotated checklist of bumble bees with an analysis of patterns of description (Hymenoptera: Apidae, Bombini). Bull. Nat. Hist. Mus. Entomol..

[bib100] Williams P.H., Brown M.J., Carolan J.C., An J., Goulson D., Aytekin A.M., Best L.R., Byvaltsev A.M., Cederberg B., Dawson R. (2012). Unveiling cryptic species of the bumblebee subgenus *Bombus s. str.* worldwide with COI barcodes (Hymenoptera: Apidae). Syst. Biodivers..

[bib101] Xu J., Strange J.P., Welker D.L., James R.R. (2013). Detoxification and stress response genes expressed in a western North American bumble bee, Bombus huntii (Hymenoptera: Apidae). BMC Genom..

[bib102] Yu G., Wang L.G., Han Y., He Q.Y. (2012). ClusterProfiler: an R package for comparing biological themes among gene clusters. OMICS A J. Integr. Biol..

[bib103] Zambra E., Martinet B., Brasero N., Michez D., Rasmont P. (2020). Hyperthermic stress resistance of bumblebee males: test case of Belgian species. Apidologie.

