# Supplementary material for: Temperature and livestock grazing trigger transcriptome responses in bumblebees along an elevational gradient
Source: iScience. 2022 Sep 22;25(10):105175. doi: 10.1016/j.isci.2022.105175 (PMC9530833; doi:10.1016/j.isci.2022.105175)
Supplement: Document S1. Figures S1–S4 [file mmc1.pdf]

iScience, Volume *25*

Supplemental information

Temperature and livestock grazing trigger transcriptome responses in bumblebees along an elevational gradient

Kristof Brenzinger, Fabienne Maihoff, Marcell K. Peters, Leonie Schimmer, Thorsten Bischler, and Alice Classen

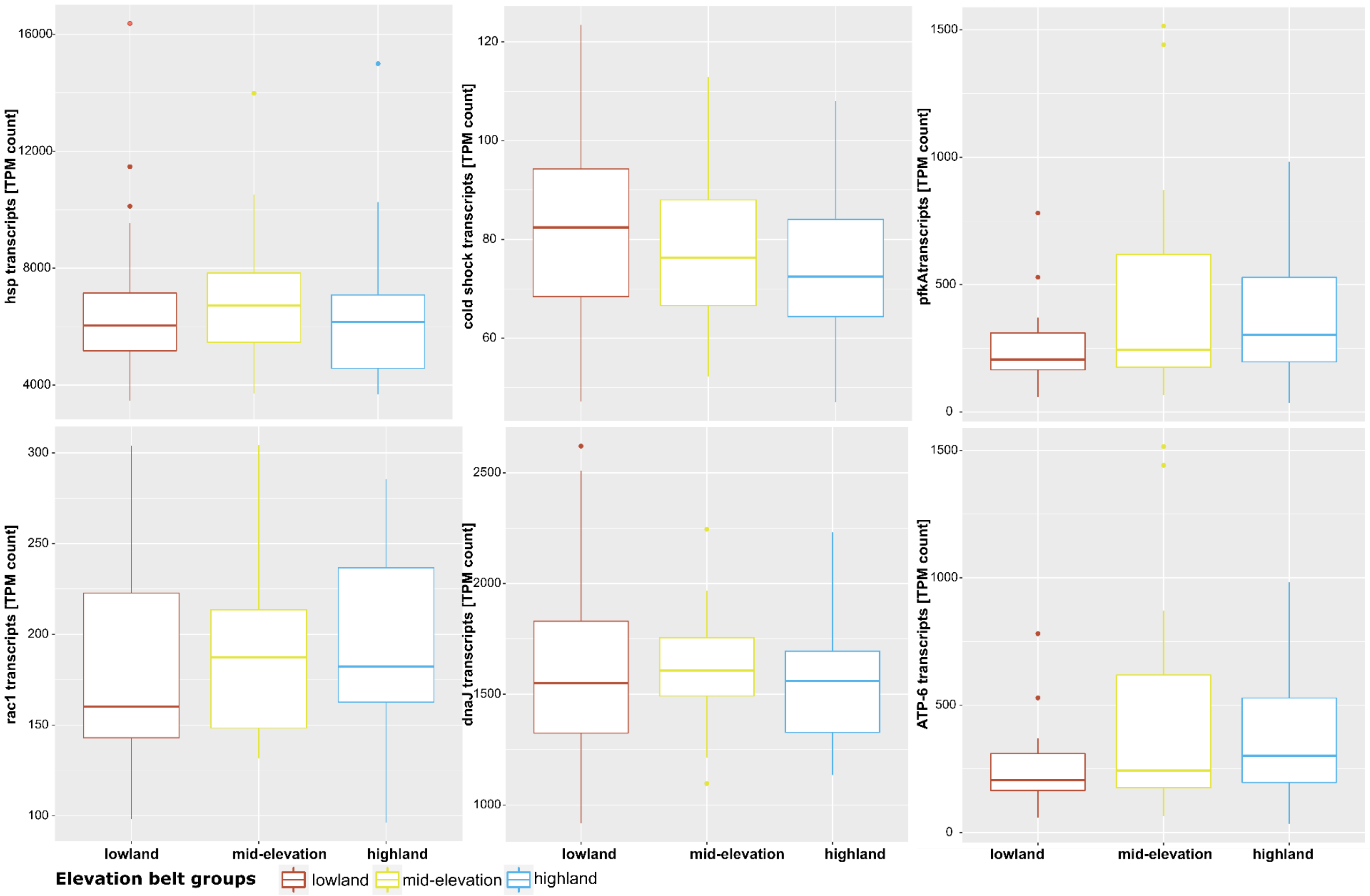

Supplementary Figure 1: Expression levels of different important genes involved in temperature or elevation stress responses, related to Figure 2. Expression levels of different important genes involved in temperature or elevation stress responses between the different elevation belts obtained from DESeq2 analysis (Part 1).

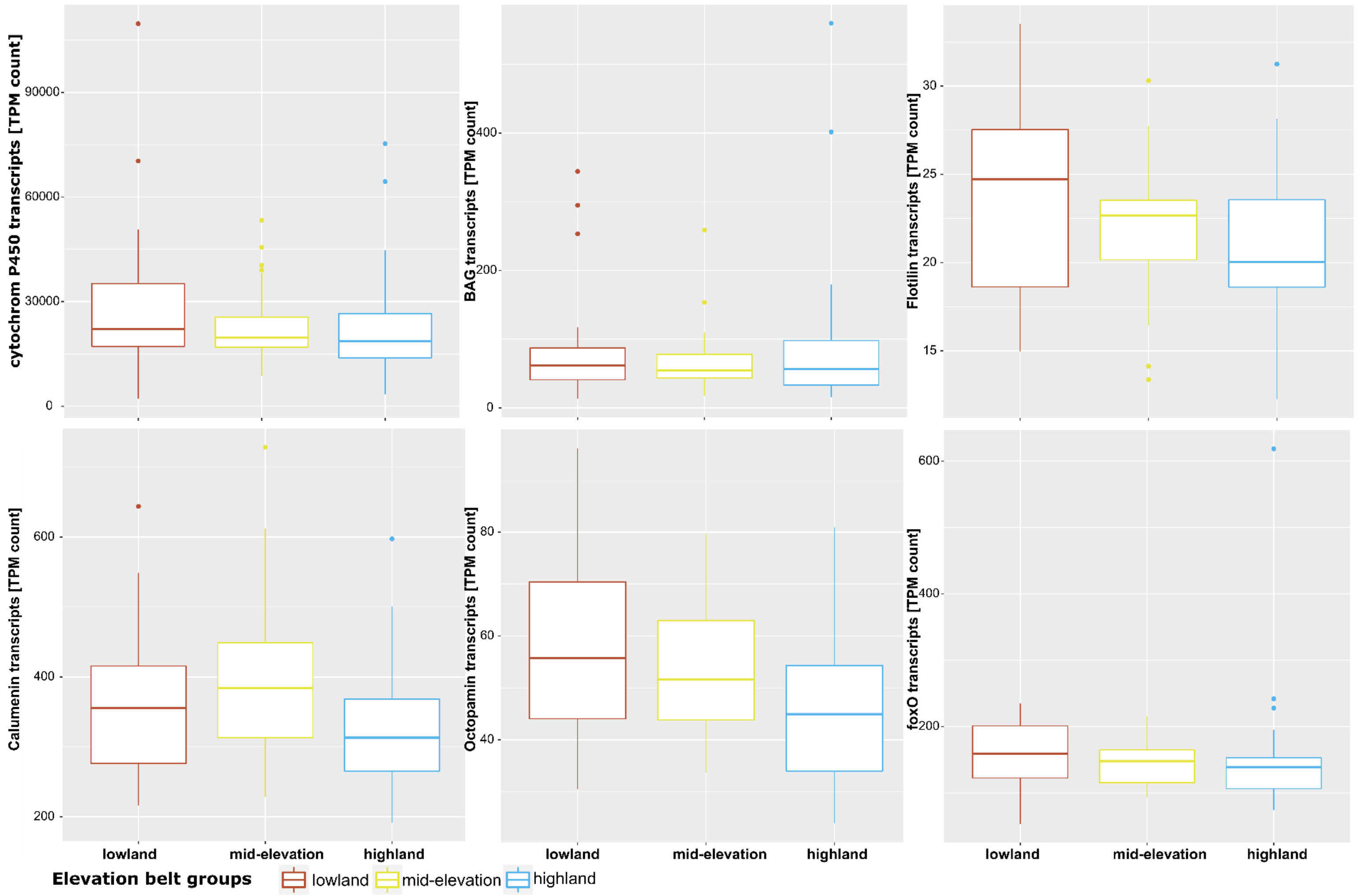

Supplementary Figure 2: Expression levels of different important genes involved in temperature or elevation stress responses, related to Figure 2. Expression levels of different important genes involved in temperature or elevation stress responses between the different elevation belts obtained from DESeq2 analysis (Part 2).

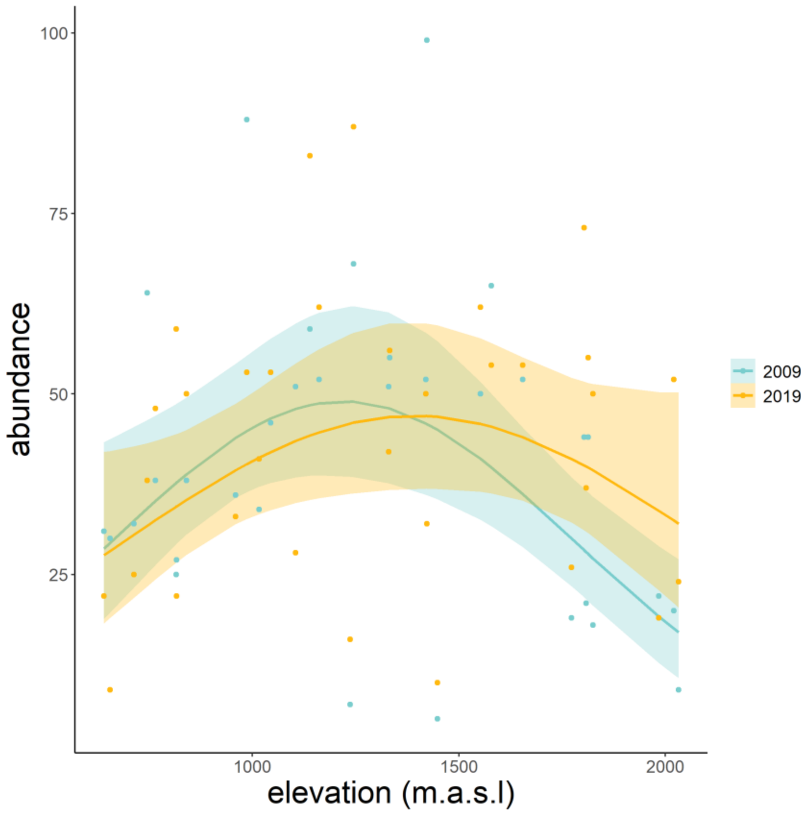

Supplementary Figure 3: Abundance of bumblebees along the elevational gradient in two sampled years 2009 and 2019, related to STAR Method. We captured the change in abundance with generalized mixed models (glmmTMB). Year and elevation were tested as interaction and individually in the model. Elevation was included as a quadratic term because there was no linear relationship between elevation and abundance. As the significant interaction term shows (interaction: X2(2)= 26.82, p <0.001***), abundance at the highest sites was higher in 2019 than in 2009.

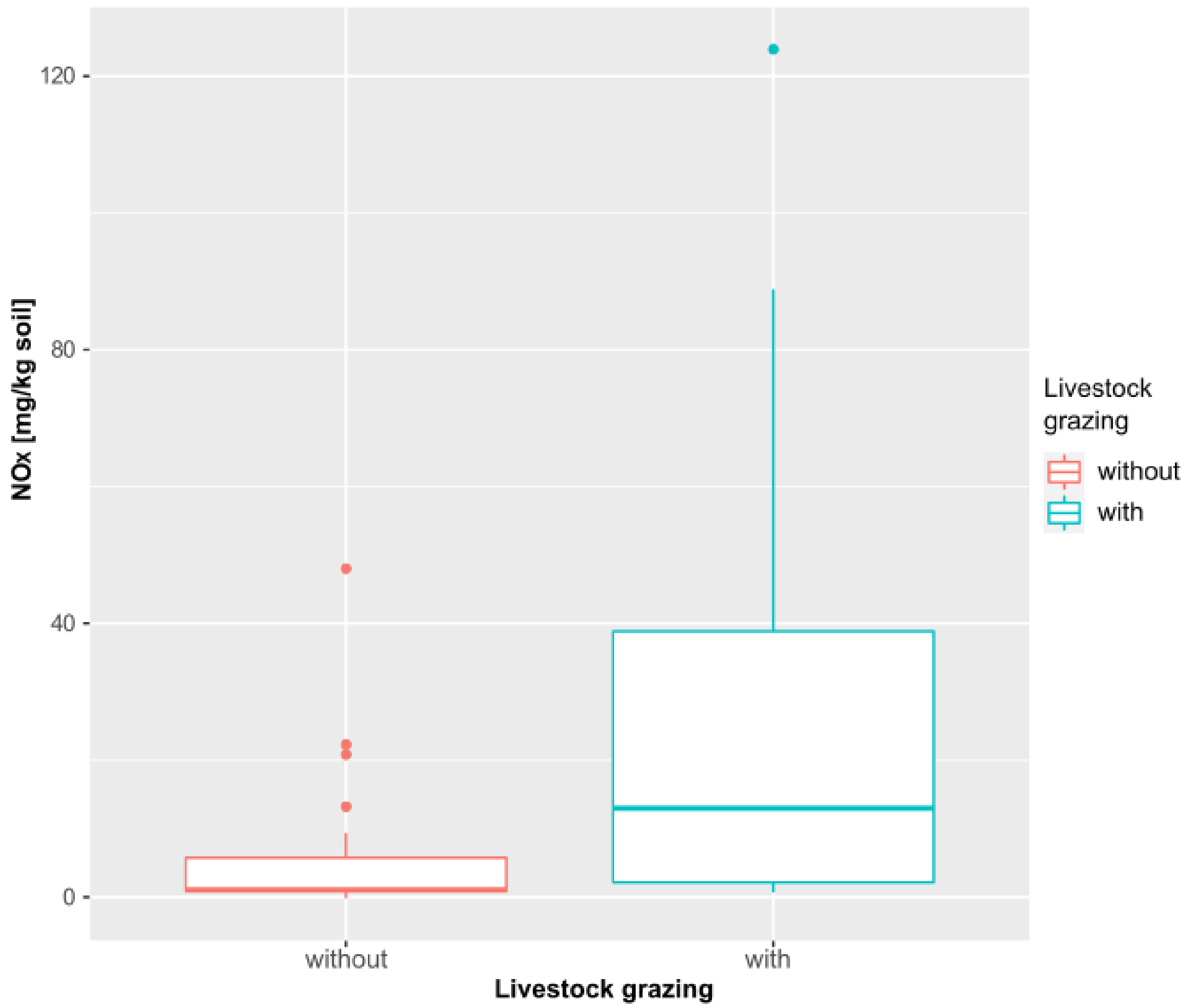

Supplementary Figure 4: NOx concentration on sampling sites with and without grazing, related to STAR Method. Concentration of NOx (NO_3+NO_2) in mg/kg soil from sampling sites with and without livestock grazing
